# SPY Interacts With Tubulin and Regulates Abscisic Acid‐Induced Stomatal Closure in Arabidopsis

**DOI:** 10.1002/pld3.70063

**Published:** 2025-04-01

**Authors:** Tongtong Liu, Pan Wang, Zixuan Wang, Weipeng Dun, Jing Li, Rong Yu

**Affiliations:** ^1^ College of Life Sciences Capital Normal University Beijing China; ^2^ State Key Laboratory of Plant Environmental Resilience, College of Biological Sciences China Agricultural University Beijing China; ^3^ Beijing Key Laboratory of DNA Damage Response Capital Normal University Beijing China

**Keywords:** Arabidopsis, *O*‐fucose, SPY, stomata movement, tubulin

## Abstract

Sugars are important both as an energy source and a signaling cue. In *Arabidopsis thaliana,* SPINDLY (SPY) is the *bona fide* *O*‐fucosylation transferase that links sugar with various plant growth and development processes. Previously, *spy* was shown to display a strong salt and drought tolerance phenotype. Herein we confirmed the phenotype and further studied its mechanism. We found that abscisic acid (ABA) elevated *SPY* expression in guard cells, and SPY is involved in ABA‐induced stomatal closure. We show that SPY regulates the rearrangement of the microtubule cytoskeleton in guard cells. Moreover, ABA‐induced microtubule reorganization is enhanced in *spy* mutants. Mechanistically, SPY interacts with α‐tubulin1 (TUA1) in both yeast‐two hybrid, bimolecular fluorescence complementation and split luciferase complementation imaging assays, indicating that TUA1 may be *O*‐fucosylated by SPY. Our work is in line with the notion that SPY has many substrates involved in diverse processes in plants, and also unearths a key mechanism how glycosylation regulates the stomata movement via the microtubule cytoskeleton.

**Abbreviations**
ABA: abscisic acid
AAL: Aleuria aurantia lectin
BiFC: bimolecular fluorescence complementation
LCI: luciferase complementation imaging
*O*‐GlcNAc: *O*‐linked β‐N‐acetylglucosamine
OGT: *O*‐GlcNAc transferase
PTM: post‐translational modification
SPY: SPINDLY
Sec: Secret Agent
YFP: yellow fluorescent protein

## 1 Introduction

Sugars are important sources of energy as well as vital signaling cues for both animals and plants. In animals, more than four decades of research has established that *O*‐linked N‐acetylglucosamine (*O*‐GlcNAc), the mono‐saccharide modification of proteins, takes part in various biological pathways, encompassing cancer, neurodegenerative diseases, immune diseases, and cellular homeostasis (Yang and Qian 2017). There is only one *O*‐GlcNAc writer in animals:*O*‐GlcNAc transferase (OGT), and there is only one eraser:the *O*‐GlcNAcase (OGA). In contrast, two OGT homologs, SPINDLY (SPY) and SECRET AGENT (SEC), have been identified in Arabidopsis (Hartweck et al. 2002), and the double mutant of *sec spy* shows synthetic lethality (Hartweck et al. 2002). Later studies demonstrate that SEC is the canonical OGT, as it shows auto‐glycosylation activity (Hartweck et al. 2002), while SPY is a *bona fide* *O*‐fucose transferase (POFUT) and mediates *O*‐fucosylation of RGA and pseudo‐response regulator 5 (PRR5) (Zentella et al. 2017; Wang et al. 2020; Sun 2021). In rice, there are two OsOGT (Li et al. 2023). And the plant OGA is yet to be identified.

The emerging chemoproteomic studies in plants are just beginning to unravel the sweet signaling pathways in the plant world. Using lectin affinity chromatography coupled with mass spectrometry, the *O*‐fucosylome is found to include vital proteins in development and phytohormone signaling in Arabidopsis (Bi et al. 2023; Zentella et al. 2023). And SEC also modifies hundreds of protein substrates in diverse pathways (Xu et al. 2017; Wu et al. 2022; Shrestha et al. 2024). In rice, the two OsOGTs mediate *O*‐GlcNAcylation on hundreds of proteins involved in transcription, translation and plant hormone signaling (Li et al. 2023). By comparing these proteomic data, a conclusion is reached that there is significant overlap between the *O*‐fucosylome and *O*‐GlcNAcome, suggesting that somehow disparate sugars will find the same targets to finetune the biological processes.

Between *sec* and *spy*, the latter has more prominent defects in plant growth, including seedlings, leaves, flowering time, circadian clock, abscisic acid (ABA) signaling, abiotic stresses, just to name a few (Sun 2021). In this study, we set out to examine the stomata movement phenotype in *spy,* as previous studies have shown a strong salt and drought tolerance phenotype (Qin et al. 2011). We found that the expression of *SPY* in guard cells is upregulated by ABA, and SPY is involved in ABA‐induced stomatal closure. Mechanistically, we show that tubulin interacts with SPY and could be *O*‐fucosylated. Moreover, SPY regulates the rearrangement of microtubules in guard cells. Our work thus identifies a potential substrate of SPY and implicates SPY in the sweet link to couple nutrient status with plant growth and survival under stress.

## 2 Results

### 2.1 The *spy* Mutant Enhances Drought Tolerance

**FIGURE 1:**
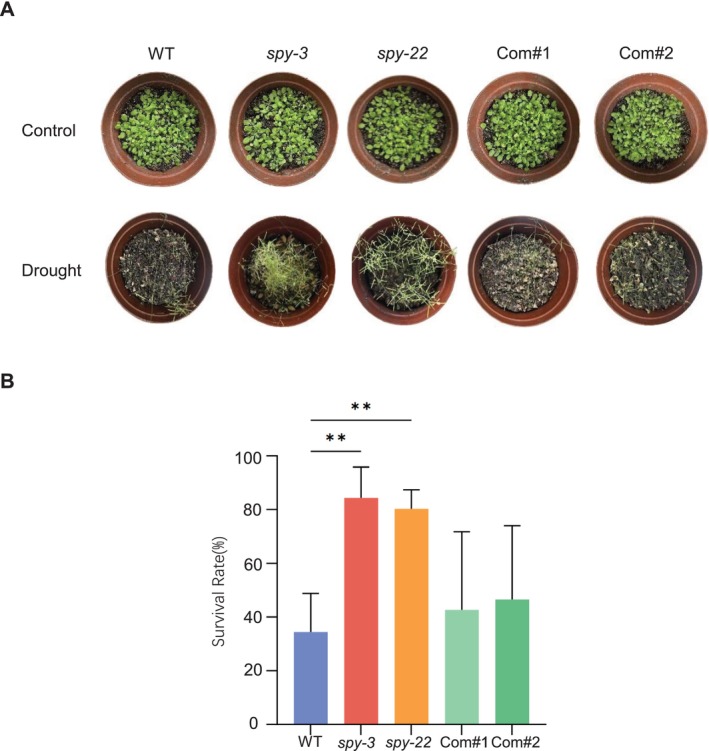
The *spy* mutant exhibited enhanced drought tolerance. (A) Drought phenotypes of different seedlings in soil. *spy‐3, spy‐22*, the two complementation lines (Com#1, Com#2), and wild‐type (WT) plants were grown on 1/2 MS medium plates for 10 days and then transferred to moist soils without further watering. The phenotypes were observed and photographed after complete drought treatment for 4 weeks. (B) The survival rate was calculated from independent experiments. Values represent mean ± SD for three independent experiments. Two‐tailed Student's *t* test, **p < 0.01.

To examine whether the *SPY* gene plays a role in the plant drought stress response, we obtained two mutant strains: the point mutation mutant *spy‐3* (Jacobsen and Olszewski 1993; Jacobsen et al. 1996) and the T‐DNA insertion mutant *spy‐22* (Mutanwad et al. 2020). The *spy‐3* mutation resulted in an amino acid substitution in the catalytic domain, while the *spy‐22* mutation caused a T‐DNA insertion in the 5′ UTR domain (Jacobsen et al. 1996; Figure S1). We then proceeded to analyze the difference in the survival of the *spy* mutants (*spy‐22* and *spy‐3*) and wild‐type (WT) plants after soil drying conditions. 10‐day‐old seedlings were transferred to moist soils without watering. After 4 weeks of complete drought treatment, the WT plants exhibited significant wilting, leaf shriveling, and crumpling. In contrast, in the *spy* mutants the majority of the leaf blades were green (Figure 1A). The statistical analysis of the data obtained in this experiment is presented in Figure 1B. The survival rates of *spy* mutants were much higher than those of WT after rewatering, indicating that *spy* mutants exhibited enhanced drought tolerance, consistent with previous reports (Qin et al. 2011). Drought phenotypes in the two complementary lines (Com#1 and Com#2) were similar to the wild type reasonably.

### 2.2 The Complement of *SPY* Suppresses the Drought Tolerance in *spy* Mutants

**FIGURE 2:**
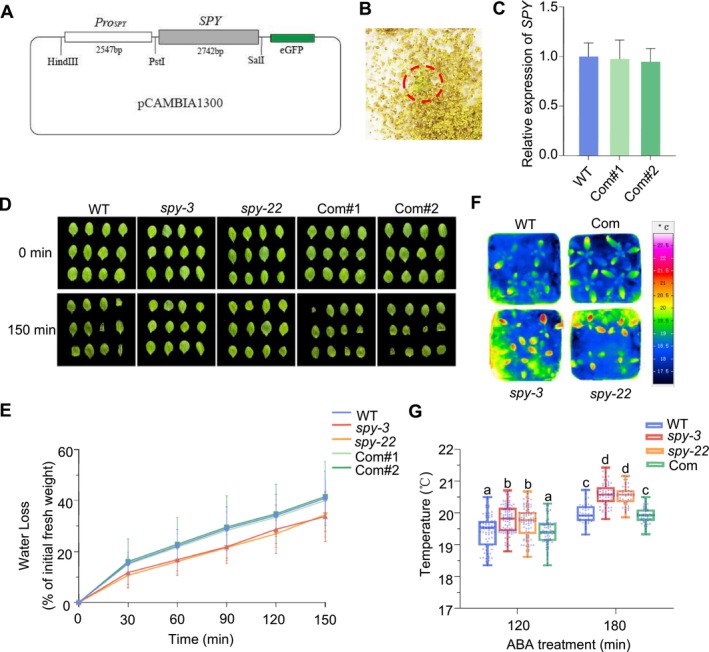
The complement of *SPY* rescues the *spy* phenotype. (A,B) Construction of the *spy‐22* mutant complemented line. (C) RT‐qPCR analysis of *SPY* gene expression in complemented lines. The expression level was standardized with the internal reference gene *EF1‐α*, and three independent biological replications were performed. The data are average ± SEM. (D,E) Fresh weights of detached leaves from different seedlings including WT, *spy* mutants, and complemented lines. Their weights were measured at 30‐min intervals. Values represent the mean ± SD for three independent experiments, each with a minimum of 12 leaves. (F) After ABA treatment, infrared thermography was obtained of seedlings from WT, mutants, and complemented lines. Leaf temperature was measured using infrared camera software. (G) The temperatures of no less than 30 leaves were measured. The box and whisker plots show minimum and maximum values, respectively. The line in the box indicates the median value, and the boundaries represent the 25th percentile (upper) and 75th percentile (lower). Different letters represent significant differences at *p* < 0.05 (one‐way ANOVA).

To further investigate the function of *SPY* in plant drought tolerance, we constructed complemented lines of the *spy‐22* mutant (Figure 2A,B), and Lines 1 and 2 were selected for further analysis. The *SPY* transcript level was significantly reduced in the *spy‐22* mutant (Figure S1C), and there was no notable difference in the expression levels between the WT and complemented lines (Com#1 and Com#2) (Figure 2C). Subsequently, we used the detached rosette leaves of 4‐week‐old plants and measured their fresh weights every 30 min to determine the rate of water loss (Figure 2D). As shown in Figure 2E, the rate of water loss was similar in WT and in complemented lines but was slower in *spy* mutants (*spy‐22* and *spy‐3*). Abscisic acid (ABA) is a plant hormone in land plants that serves to prevent water loss and to respond to drought stress. We also compared the leaf temperature of 4‐week‐old plants after ABA treatment by using an infrared camera (Figure 2F). There was no significant difference in leaf temperature between WT and complemented lines (Com); however, leaf temperatures were significantly higher in *spy* mutants than in WT (Figure 2G), and this result is in agreement with the water loss assay. Our results indicate that complementation of *SPY* suppresses the drought tolerance phenotype in *spy* mutants.

### 2.3 *SPY* Expression in Guard Cells Is Up‐Regulated by ABA

**FIGURE 3:**
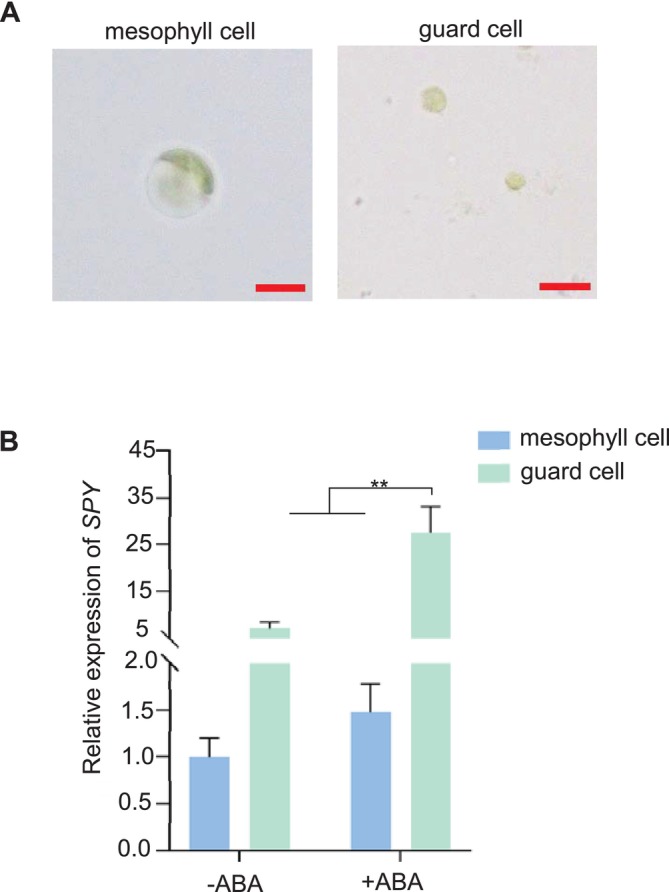
*SPY* expression in guard cells is up‐regulated by ABA. (A) Images of Arabidopsis leaf mesophyll cells and guard cell protoplasts (scale bar = 20 μm). (B) The relative expression of *SPY* in mesophyll cell protoplasts and guard cell protoplasts before and after 10‐μM ABA treatment. The expression level was standardized with the reference *EF1‐α* gene, and three independent biological replicates were performed with mean ± SEM data (two‐tailed *t* test, **p < 0.01).

As we all know, the phytohormone ABA is triggered by drought stress. It promotes stomatal closure, and decreases water loss (Kuromori et al. 2018; Bharath et al. 2021). To analyze whether the drought phenotype of the *spy* mutants is triggered by ABA signaling, we isolated the *Arabidopsis* leaf mesophyll cell protoplasts and guard cell protoplasts separately (Figure 3A) and extracted the corresponding protoplast RNA to be reverse transcribed into cDNA for RT‐qPCR. As shown in Figure 3B, the expression of *SPY* in guard cells was largely increased by ABA treatment, while there was no significant difference in the expression of SPY in leaf mesophyll cells before and after treatment under the same conditions. Collectively, these results demonstrate that the expression of the *SPY* gene was more sensitive and up‐regulated by ABA in guard cells.

### 2.4 SPY Is Involved in ABA‐Induced Stomatal Closure

**FIGURE 4:**
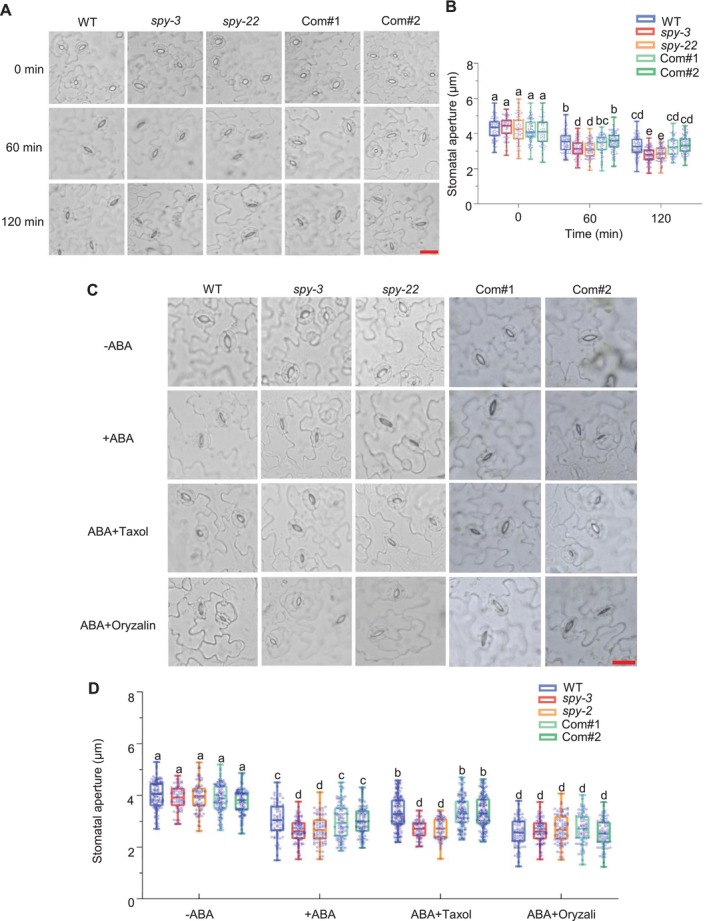
SPY is involved in ABA‐induced stomatal closure. (A) Representative images of stomatal aperture from WT, *spy* mutants, and complemented lines (scale bar = 20 μm). (B) Quantification of stomatal aperture. Detached rosette leaves from different seedlings were incubated in MES buffers for 2 h and treated with 10 μM ABA for 1 and 2 h, respectively. Stomatal aperture was measured and analyzed in at least 70 stomata using an inverted microscope. Different letters represent significant differences *p* < 0.05 (one‐way ANOVA). (C) Representative stomata in WT, mutants and complemented lines treated with microtubule drugs (Taxol and Oryzalin) (scale bar = 20 μm). (D) Statistics of stomatal apertures. Detached rosette leaves from different seedlings were incubated in MES buffers for 2 h and treated with 10 μM ABA, ABA plus 30 μM Taxol, and ABA plus 10 μM Oryzalin for 2 h. Stomatal aperture was measured and analyzed. At least 60 stomatal pores were measured in each sample. Different letters represent significant differences, *p* < 0.05 (one‐way ANOVA).

Considering that ABA‐induced stomatal closure is a common response to drought stress in plants, we further investigated the changes in the stomatal aperture of WT, *spy* mutants, and complemented lines. We cut the rosette leaves of 4‐week‐old plants and incubated the leaves in an MES buffer with cold light irradiation for 2 h to completely open the stomata, then treated them with 10 μM ABA and subject it to microscopy (Figure 4A). The mean stomatal apertures of all plants decreased correspondingly. However, the *spy* mutants (*spy‐22* and *spy‐3*) were more sensitive to ABA treatment and exhibited a higher stomatal closure compared with WT, and there was no significant difference between the complemented lines (Com#1 and Com#2) and the WT (Figure 4B). These results demonstrate that SPY participates in regulating ABA‐induced stomatal closure in response to drought stress.

In addition, microtubule disassembly plays an important role in ABA‐induced stomatal closure. When stomata are in an open state, the microtubules exhibit a well‐organized radial pattern along the ventral to dorsal walls in guard cells; this pattern undergoes disassembly as the stomata close and reassemble as they open (Eisinger et al. 2012; Jiang et al. 2014; Dou et al. 2021; Wang et al. 2023). We further measured the stomatal aperture of all plants treated with ABA plus microtubule‐specific drugs (ABA plus Taxol or ABA plus Oryzalin). As shown in Figure 4C, the *spy* mutants were less sensitive to both drugs in comparison to WT (Figure 4D). Collectively, these results indicated that the *SPY* mutation may influence the microtubule dynamics of guard cells in ABA‐induced stomatal movement.

### 2.5 SPY Interacts With TUA1

**FIGURE 5:**
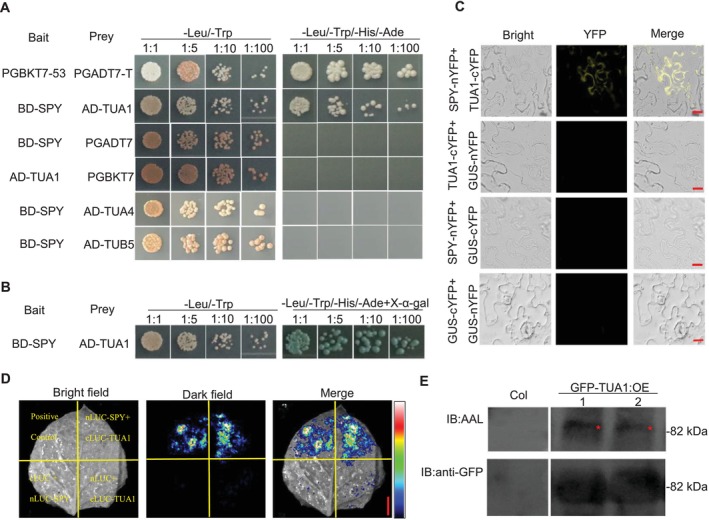
SPY interacts with TUA1 (A,B). Yeast two‐hybrid assays showing that TUA1 interacts with SPY. Yeast clones were grown on synthetic dropout (SD) medium lacking Leu and Trp for initial screening, before being transferred to SD medium lacking Leu, Trp, His, and Ade, and X‐α‐Gal (B). Yeast cultures were serially diluted as indicated. (C) SPY interacts with TUA1 in the Bimolecular Fluorescence Complementation (BiFC) assay. Leaves of *N. benthamiana* were transformed with indicated construct pairs. They were incubated for 2 days and then observed under a multifunctional fluorescence microscope. Signals were detected in leaves co‐transformed with TUA1 and SPY (scale bar = 20 μm). (D) The firefly Luciferase Complementation Imaging (LCI) assay in *N. benthamiana* leaves. Leaves of *N. benthamiana* were transformed with indicated construct pairs, incubated for 2 days, and then sprayed with reaction substrates, and the fluorescence signal was observed with a cooled CCD. The pseudo‐color bar shows the range of luminescence (scale bar = 1 cm). (E) TUA1 is *O*‐fucosylated. Total proteins were extracted from WT and TUA1 overexpressing *Arabidopsis* plants. The precipitated proteins were analyzed by immunoblotting using anti‐GFP and Aleuria Aurantia Lectin (AAL) antibodies, respectively.

As the results above suggest that SPY may regulate microtubule dynamics, we sought to detect the direct interaction between SPY and tubulin. We selected tubulin paralogs (TUA1, TUA4, and TUB5) *in Arabidopsis* according to Ji et al.'s study on glycosylation modification of tubulin in animals (Ji et al. 2011) and performed yeast two‐hybrid assays. We found that SPY specifically interacts with TUA1, but not with TUA4 or TUB5 (Figure 5A,B).

To test the interaction in vivo, we performed a bimolecular fluorescence complementation (BiFC) assay and a split luciferase complementation imaging assay (LCI) in *N. benthamiana* leaves. In the BiFC assay, the yellow fluorescent protein (YFP) reconstitution signal was observed when the fusion constructs *nYFP‐SPY* and *cYFP‐TUA1* were present (Figure 5C). And the luminescence signal was detected when *nLUC‐SPY* was co‐transformed with *cLUC‐TUA1* in the LCI assay (Figure 5D). These results demonstrate the specific interaction between SPY and TUA1.

Moreover, we generated transgenic *Arabidopsis* seedlings overexpressing TUA1‐GFP in the WT backgrounds and extracted the total proteins of the WT and TUA1 overexpressing plants, which were then analyzed by immunoblotting using anti‐GFP and Aleuria aurantia lectin (AAL), respectively. As shown in Figure 5E, we found that in the same position of GFP‐TUA1, GFP‐TUA1 was specifically recognized by AAL, which binds specifically to the terminally attached fucose (Wang et al. 2020; Bi et al. 2023; Zentella et al. 2023). Together, these results demonstrate that TUA1 interacts with SPY and might be *O*‐fucosylated.

### 2.6 SPY Regulates the Rearrangement of Microtubules in Guard Cells

**FIGURE 6:**
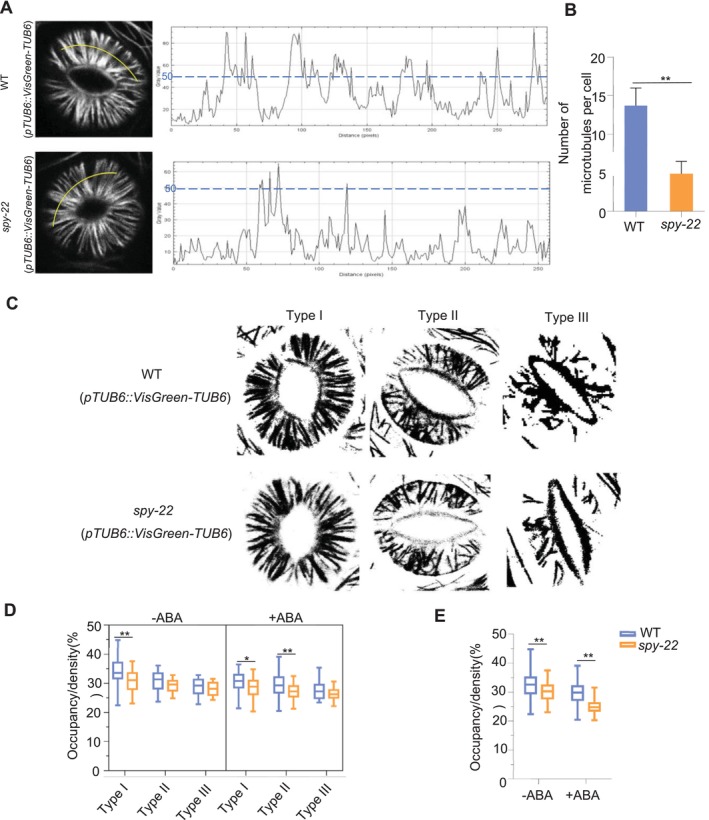
SPY regulates the rearrangement of microtubules in guard cells. Type I:microtubules exhibit a well‐organized radial pattern along the ventral to dorsal walls of the cell and are commonly found in guard cells with open stomata. Type II: Microtubules in a radial pattern are reduced, with varying degrees of crossing, tilting, and interlacing into a web‐like arrangement. Type III: Many microtubules were disrupted and disappeared in guard cells, mostly found in closed stomata. (A,B) Continuous fluorescent intensity of the GFP signal along the line in guard cells with Type I microtubule orientation was measured in Col (*pTUB6::VisGreen‐TUB6*) and *spy‐22* (*pTUB6::VisGreen‐TUB6*). Filament numbers of Type I were determined by the number of fluorescent peaks with an intensity higher than 50 (Gray value) (*n* ≥ 60, mean ± SEM, two‐tailed *t* test, **p < 0.01). *spy‐22* (*pTUB6::VisGreen‐TUB6*) plants were generated by crossing. (C) Skeletonized microtubules in guard cells from Col (*pTUB6::VisGreen‐TUB6*) and *spy‐22* (*pTUB6::VisGreen‐TUB6*) by Image J software. (D) The occupancy of three types (Type I/ Type II/Type III) of skeletonized microtubules in guard cells before or after 10‐μM ABA treatment (*n* ≥ 100, two‐tailed *t* test, *p < 0.05, **p < 0.01). (E) The occupancy of microtubules in guard cells before or after ABA treatment (*n* ≥ 100, two‐tailed *t*‐test, *p < 0.05, **p < 0.01).

The distribution of microtubules in guard cells is classified into three types (Fukuda et al. 1998). The radial state (Type I): Microtubules exhibit a well‐organized radial pattern along the ventral to dorsal walls of the cell and are commonly found in guard cells with open stomata. The transitional state (Type II): Microtubules in a radial pattern are reduced, with varying degrees of crossing, tilting, and interlacing into a web‐like arrangement. The fragmental state (Type III): Many microtubules were disrupted and disappeared in guard cells, mostly found in closed stomata. To further confirm the function of SPY in ABA‐induced microtubule disassembly, we crossed the *spy‐22* mutant with *pTUB6::VisGreen‐TUB6* in the Col background plants to observe and quantify GFP‐tubulin‐labeled microtubules in the guard cells. As shown in Figure 6A, according to the measurement of the continuous fluorescent intensity of the GFP signal along the line in guard cells with Type I microtubule orientation, we found that the microtubules exhibited well‐organized radial filaments in both the Col (*pTUB6::VisGreen‐TUB6*) and *spy‐22* (*pTUB6::VisGreen‐TUB6*) guard cells when the stomata were in the open state, but the filament number of fluorescent peaks with a gray value greater than 50 was greatly reduced in *spy‐22* (*pTUB6::VisGreen‐TUB6*) (Figure 6B).

Subsequently, we then skeletonized microtubules in guard cells using Image J software (Figure 6C) and measured the occupancy of microtubules to assess the density of microtubules (Dou et al. 2021; Wang et al. 2023). Along with ABA‐induced stomatal closure, the density of microtubules in Col (*pTUB6::VisGreen‐TUB6*) guard cells was reduced in the presence of ABA, and the microtubule density decreased significantly in *spy‐22* (*pTUB6::VisGreen‐TUB6*) guard cells than those of Col (*pTUB6::VisGreen‐TUB6*) (Figure 6, D and E), which is consistent with the confocal observation. Taken together, these findings demonstrate that SPY regulates the rearrangement of microtubules in guard cells.

### 2.7 ABA‐Induced Microtubule Reorganization Is Enhanced in *spy* Mutants

**FIGURE 7:**
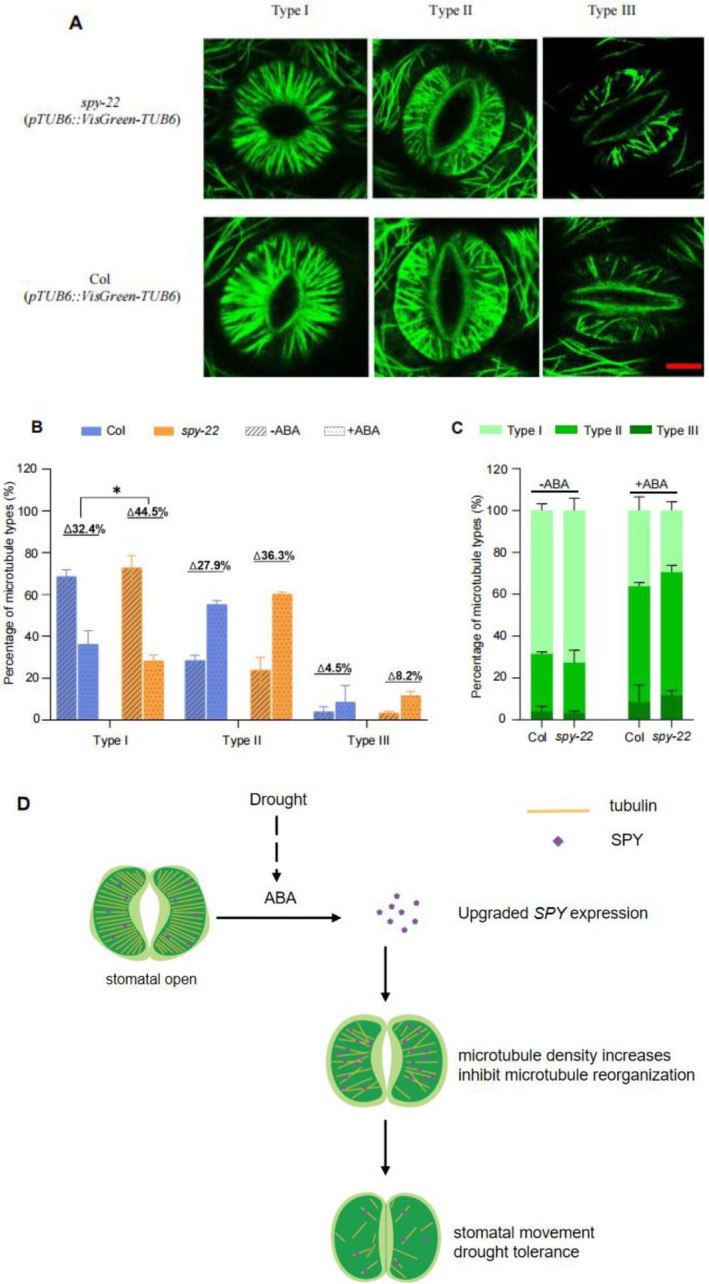
ABA‐induced microtubule reorganization is enhanced in *spy* mutants. (A) Microtubules in guard cells from Col (*pTUB6::VisGreen‐TUB6*) and *spy‐22* (*pTUB6::VisGreen‐TUB6*) were treated with 10 μM ABA. Images show typical patterns (Type I/ Type II/Type III) (scale bar = 5 μm). (B) Statistical analysis of the proportion‐changes with different microtubule pattern types in response to ABA treatment (Student's *t* test, *p < 0.05.) (C) Proportions of different microtubule arrangement types in guard cells of Col (*pTUB6::VisGreen‐TUB6*) and *spy‐22* (*pTUB6::VisGreen‐TUB6*) before or after ABA treatment. Values represent the mean ± SD for three independent experiments, with over 200 guard cells measured in total. (D) Proposed working model of SPY‐catalyzed tubulin *O*‐fucose modification in regulating ABA‐induced microtubule dynamic transitions and stomatal closure in *Arabidopsis*.

To study the underlying mechanism of SPY‐mediated microtubule reorganization in response to ABA in guard cells, we observed the microtubule reorganization of Col (*pTUB6::VisGreen‐TUB6*) and *spy‐22* (*pTUB6::VisGreen‐TUB6*) guard cells (Figure 7A). Confocal microscopy showed that after ABA treatment, the well‐organized microtubule filaments were largely disrupted in both Col (*pTUB6::VisGreen‐TUB6*) and *spy‐22* (*pTUB6::VisGreen‐TUB6*) guard cells. However, the microtubule dynamic transitions in the *spy‐22* (*pTUB6::VisGreen‐TUB6*) mutant guard cells were much more rapidly regulated compared with the Col (*pTUB6::VisGreen‐TUB6*), especially that the Type I microtubule pattern decreased in *spy‐22* (*pTUB6::VisGreen‐TUB6*) significantly more than in Col (*pTUB6::VisGreen‐TUB6*) (Figure 7B,C). These results indicate that ABA‐induced microtubule reorganization is enhanced in *spy* mutants.

## 3 Discussion

In this work, we examined the drought tolerance phenotype in *SPY,* and found that *SPY* expression in guard cells is up‐regulated by ABA, and SPY‐catalyzed tubulin *O*‐fucose modification is critical for ABA‐induced microtubule reorganization and stomatal closure in response to drought stress (Figure 7D). The *spy* mutant was shown to have a strong salt and drought tolerance phenotype previously (Qin et al. 2011), and our work provided a mechanistic insight that SPY may exert its effect via the tubulin cytoskeleton network.

Intriguingly, about half of *O*‐GlcNAcylated proteins are *O*‐fucosylated (Bi et al. 2023). Indeed, SPY itself is subject to both *O*‐GlcNAcylation and *O*‐fucosylation. Tubulin was shown to be *O*‐GlcNAcylated in animals (Walgren et al. 2003; Ji et al. 2011). Due to the technical barriers at that time, no exact modification sites were identified. Recently, TUA4, TUA3, TUA6 were identified in an *O*‐fucosyl profiling work (Bi et al. 2023), although TUA1 was not directly detected in the proteomics, suggesting that other tubulin paralogs besides TUA1 may also be *O*‐fucosylated.

As a newly identified POFUT, identification of SPY substrates and elucidation of their functions will open up new venues for understanding how sugar integrates various biotic and abiotic stresses to plant growth and survival. DELLA, for instance, is known to be *O*‐GlcNAcylated by SEC (Zentella et al. 2016), and only later found to be also *O*‐fucosylated by SPY (Zentella et al. 2017). Moreover, these two glycosylation events antagonize each other in regulating DELLA activity.

Another example is AtACINUS, a regulator of both transcription and RNA splicing. It is also both *O*‐GlcNAcylated and *O*‐fucosylated (Bi et al. 2021), and SPY and SEC are found to modulate Acinus‐dependent intron splicing. But its human homolog ACIN1 has not been linked with glycosylation, suggesting that studying glycosylation in the plant world will also provide new insights into animals.

Tubulins are pivotal for many biological processes, such as cell division, trafficking, and cell shape regulation. It is no wonder that it is subject to multifaceted regulations. For example, when facing excess soluble tubulin, co‐translational degradation of nascent tubulin mRNAs is trigged by tetratricopeptide protein 5 (TTC5), a tubulin‐specific ribosome‐associating factor (Lin et al. 2020). In long‐lived microtubules in neurons, tubulins manifest both detyrosination (Yu et al. 2021) and polyglutamylation (Genova et al. 2023). Chemical semi‐synthesis of modified tubulin further revealed that the polyglutamylation cycle directs detyrosination (Ebberink et al. 2022). If we could map *O*‐fucosylation sites on tubulins in the future, we may follow suit and perform the same chemical engineering to dissect the exact role of glycosylation on the microtubule cytoskeleton.

## 4 Experimental Procedures

### 4.1 Plant Materials and Growth Conditions

*Arabidopsis thaliana* Columbia (Col‐0) was used as the WT ecotype. After surface sterilization with 70% ethanol, the seeds were sown and germinated on 1/2 Murashige and Skoog medium (2.2 g/L MS salts, 10 g/L sucrose, pH 5.6, 8 g/L agar) for 10 days. The seedlings were transferred into soil and were grown under a 16‐h light/8‐h dark photoperiod in a growth room at 22°C. *N*. *benthamiana* seeds were germinated in soil, and seedlings were grown for 3–4 weeks under the same conditions as *Arabidopsis*. T‐DNA insertional mutants *spy‐22* (SALK_090582) (Mutanwad et al. 2020) and point mutants *spy‐3* (Jacobsen and Olszewski 1993; Jacobsen et al. 1996) and previously published reporter lines *pTUB6::VisGreen‐TUB6* (Liu et al. 2019) in Col background were used. For overexpressing transgenic plants, the coding sequence of TUA1 was amplified by PCR and inserted into *pCAMBIA1300‐GFP* vector between *Pst* I and *Sal* I sites. To complement the phenotype of the *spy‐22* mutant, the coding sequence and the promoter of SPY were amplified by PCR and inserted into *pCAMBIA1300‐GFP* vector under the control of the SPY promoter between *Pst* I and *Sal* I sites. The *spy‐22* (*pTUB6::VisGreen‐TUB6*) was generated in this study by genetically crossing *pTUB6::VisGreen‐TUB6* and *spy‐22* mutants. The primers are shown in Supplemental Tables S1–S4.

### 4.2 Plasmid Construction

For yeast two‐hybrid assays, the coding sequences of TUA1, TUA4, and TUB5 were amplified by PCR and separately inserted into the *pGADT7* vector between *EcoR* I and *BamH* I sites. The coding sequence of SPY was amplified by PCR and inserted into the *pGADT7* vector between *BamH* I and *Pst* I sites. The primers are shown in Table S6.

For bimolecular fluorescence complementation (BiFC) assay, the coding sequence of SPY was amplified by PCR and inserted into *pCAMBIA‐1300‐nYFP* vector between *Xbal* I and *BamH* I sites. The coding sequence of TUA1 was amplified by PCR and inserted into *pCAMBIA‐1300‐cYFP* vector between *Xbal* I and *BamH* I sites. The primers are shown in Table S7.

For firefly Luc complementation imaging (LCI) assay, the coding sequence of SPY was amplified by PCR and inserted into *pCAMBIA‐nLUC* vector between *Kpn* I and *Sal* I sites. The coding sequence of TUA1 was amplified by PCR and inserted into *pCAMBIA‐cLUC* vector between *Kpn* I and *Sal* I sites. The primers are shown in Table S8.

### 4.3 Drought Treatment and Water Loss Assays

For the drought treatment, 10‐day‐old *Arabidopsis* seedlings were transferred to pots containing the same amount of soil and water, withholding water for 4 weeks under normal conditions in a growth room under 16‐h light/8‐h dark cycle conditions. Plants that maintained well‐watered conditions were used as a control.

For water loss assay, rosette leaves were cut from 4‐week‐old plants and placed on a laboratory bench. The weight of the detached leaves was measured every 0.5 h for 2.5 h. Water contents were expressed as the percentage of fresh weight.

### 4.4 Stomatal Aperture Measurement

Stomatal aperture assays were performed as described (Dou et al. 2021; Wang et al. 2023). The rosette leaves of 4‐week‐old plants in soil were incubated in MES buffer (50 mM KCl, 10 mM CaCl_2_, and 10 mM MES, pH = 6.15) in a growth chamber for 2 h to completely open the stomata. Then, the leaves were transferred to MES buffer containing 10 μM ABA plus 0 or microtubule‐specific drugs (30 μM Taxol or 10 μM Oryzalin) for the indicated time periods. Epidermal strips after different treatments were photographed with a ZEISS Axiovert 200 M microscope. Stomatal apertures were measured with ImageJ software.

### 4.5 Infrared Thermograph Imaging

Thermal imaging was used to monitor leaf temperature as described (Wang et al. 2023). Four‐week‐old plants in soil were treated with 10 μM ABA for the indicated time periods. Then photographs were taken with a thermal imaging camera (VarioCAM HD head 880), and the leaf temperatures were measured using InfraTec GmbH IRBIS 3 analysis software (http://www.infratec.cn).

### 4.6 RNA Extraction and RT‐qPCR

Total RNA was extracted by using an RNAprep pure Plant Kit (TIANGEN, #DP432) and treated with RNase‐free DNase1 (TIANGEN, 4992232). Then it was subjected to reverse transcription with PrimeScript™ II 1st Strand cDNA Synthesis Kit (Takara, 6210A). RT‐qPCR was performed by using SYBR Premix Ex‐Taq (Takara, RR420A) in an CFX96 Real‐Time System (BIO‐RAD). The primers used are listed in Table S5.

### 4.7 Confocal Laser Scanning Microscopy

Microtubules in guard cells were observed using a confocal laser scanning microscope (Leica TCS SP8 X). The rosette leaves were incubated in MES buffers under constant light for 2 h to open the stomata completely. They were then transferred to MES buffers containing 10 μM ABA for 2 h. GFP was excited at 488 nm.

### 4.8 Yeast Two‐Hybrid Assay

The prey plasmid *pGBKT7‐SPY* was co‐transformed with the bait plasmids, such as *pGADT7‐TUA1*, *pGADT7‐TUA4*, and *pGADT7‐TUB5*, respectively, into the yeast strain AH109. The co‐transformed yeast cells were cultured on synthetic dropout (SD) medium without Trp and Leu (−TL) and SD medium without Trp, Leu, His, and Ade (‐TLHA) containing X‐gal for 3 days at 28°C. The primers used for the yeast‐two hybrid assay are listed in Table S6.

### 4.9 Bimolecular Fluorescence Complementation (BiFC) Assay

The coding sequence of SPY was cloned into *pCAMBIA‐1300‐nYFP* vectors, and the coding sequence of TUA1 was cloned into *pCAMBIA‐1300‐cYFP* vectors. The plasmids were transformed into the *Agrobacterium tumefaciens* strain GV3101, which was injected into the leaves of *N. benthamiana*. After 3 days of cultivation in a growth room, the signals of yellow fluorescent protein (YFP) were observed under a microscope (Axio Imager M2). The primers are shown in Table S7.

### 4.10 Firefly Luciferase Complementation Imaging (LCI) Assay

The coding sequence of SPY was cloned into *pCAMBIA‐nLUC* vectors, and the coding sequence of TUA1 was cloned into *pCAMBIA‐cLUC* vectors. The plasmids were transformed into the *A. tumefaciens* strain GV3101, which was injected into the leaves of *N. benthamiana*. After 2 days of cultivation in a growth room, Luciferase (LUC) signals were captured by a cooled charge‐coupled device (CCD) camera (Tanon 5200 Multi). The primers are shown in Table S8.

### 4.11 Extraction of Total Proteins and Immunoblot Analyses

Total proteins were isolated from 10‐day‐old Arabidopsis plants using protein extraction buffers [10 mM Tris–HCl, 300 mM NaCl, 2 mM EDTA, 10% (vol/vol) glycerol, 1% (vol/vol) Nonidet P‐40, 1% Triton X‐100, 1 mM PMSF] after being grounded into powder in liquid nitrogen and incubated at 4°C for 30 min. After centrifuging at 12000 g for 20 min at 4°C, the reactions were stopped with 5 × sodium dodecyl sulfate (SDS) sample buffers. Protein samples were analyzed by SDS polyacrylamide gel electrophoresis (SDS‐PAGE) and immunoblotted using the appropriate antibodies. GFP‐tagged TUA1 was detected by Western blotting using GFP (LABLEAD, G1002) and Aleuria aurantia lectin (AAL) antibodies (Vector Labs, B‐1395‐1).

### 4.12 Preparation of Protoplasts

For the isolation of guard cell protoplasts, the rosette leaves of 3–4‐week‐old *Arabidopsis* plants were cut into thin strips. These strips were then immersed in an enzyme solution (1.5% Cellulase R‐10, 0.4% Macerozyme R‐10, 0.1% BSA, 10 mM CaCl_2_, 20 mM KCl, 20 mM MES, 0.4 mM Mannitol, 50 mM β‐Mercaptoethanol, pH = 5.5) and agitated at 26°C in the dark and 60 rpm for 2 h. The enzyme solution was then mixed with W5 (2 mM MES, 0.9% NaCl, 125 mM CaCl_2_, 5 mM KCl, pH = 5.7) in equal volumes, filtered through 220 μm and 20‐μm diameter filters, and the resulting filtrate was centrifuged at 4000 rpm for 10 min, after which the supernatant was discarded. This final step was repeated once. Then W5 was added to the solution, which was incubated in an ice bath for 30 min. The guard cell protoplasts were collected by centrifugation at 1000 g for 5 min. For the isolation of mesophyll cell protoplasts, the rosette leaves of 3‐ to 4‐week‐old *Arabidopsis* plants were removed from the leaf blade epidermis with the aid of adhesive tapes. The remaining tissue was then immersed in the enzyme solution and agitated at 26°C in the dark and 60 rpm for 2 h. The enzyme solution was then mixed with W5 in equal volumes, filtered through a 220‐μm diameter filter, and subjected to the same steps as the isolation of guard cell protoplasts.

## Author Contributions

**Tongtong Liu:** investigation. **Pan Wang:** investigation. **Zixuan Wang:** investigation. **Weipeng Dun:** investigation. **Jing Li:** funding, conceptualization, resources, supervision, writing. **Rong Yu:** funding, conceptualization, data curation, supervision, project administration, writing.

## Conflicts of Interest

The authors declare no conflicts of interest.

## Supporting information


**Figure S1** Identification of *spy* mutants. A Schematic structure of the SPY protein and the locations of the mutations in the mutants B. DNA electrophoretogram of the *spy‐3* mutant (left) and sequencing result (right). Primers (F, R) were designed on both sides of the mutation site, and the target bands (240 bp) were amplified by PCR and then sequenced. C DNA electrophoretogram of the *spy‐22* mutant and RT‐qPCR analysis (right). (M:marker molecular weight is 2000 bp, mutants 1 and 2 are homozygous lines). RT‐qPCR detection of *SPY* gene mRNA expression level was standardized with internal reference gene *EF1‐α*, and three independent biological replications were performed. Data are mean ± SEM.

**Table S1** Primers for identification of *spy‐22* (SALK_090582) mutants.
**Table S2** Primers for identification of *spy‐3* mutants
**Table S3** Primers for construction of *spy‐22* complemented lines
**Table S4 ** Primers for construction of TUA1 overexpressing transgenic plants
**Table S5 ** Primers for RT‐qPCR
**Table S6 ** Primers for yeast two‐hybrid assay
**Table S7** Primers for bimolecular fluorescence complementation (BiFC) assay
**Table S8** Primers for firefly luciferase complementation imaging (LCI) assay

## Data Availability

All data are contained in this manuscript.

## References

[pld370063-bib-0001] Bharath, P. , S. Gahir , and A. S. Raghavendra . 2021. “Abscisic Acid‐Induced Stomatal Closure: An Important Component of Plant Defense Against Abiotic and Biotic Stress.” Frontiers in Plant Science 12: 615114. 33746999 10.3389/fpls.2021.615114 PMC7969522

[pld370063-bib-0002] Bi, Y. , Z. Deng , W. Ni , et al. 2021. “ *Arabidopsis* ACINUS Is *O*‐Glycosylated and Regulates Transcription and Alternative Splicing of Regulators of Reproductive Transitions.” Nature Communications 12: 945. 10.1038/s41467-021-20929-7 PMC7878923 33574257

[pld370063-bib-0003] Bi, Y. , R. Shrestha , Z. Zhang , et al. 2023. “SPINDLY Mediates *O*‐Fucosylation of Hundreds of Proteins and Sugar‐Dependent Growth in *Arabidopsis* .” Plant Cell 35: 1318–1333. 36739885 10.1093/plcell/koad023 PMC10118272

[pld370063-bib-0004] Dou, L. , K. He , J. Peng , X. Wang , and T. Mao . 2021. “The E3 Ligase MREL57 Modulates Microtubule Stability and Stomatal Closure in Response to ABA.” Nature Communications 12: 2181. 10.1038/s41467-021-22455-y PMC8041845 33846350

[pld370063-bib-0005] Ebberink, E. , S. Fernandes , G. Hatzopoulos , et al. 2022. “Tubulin Engineering by Semi‐Synthesis Reveals That Polyglutamylation Directs Detyrosination.” Nature Chemistry 15: 1179–1187. 10.1038/s41557-023-01228-8 37386282

[pld370063-bib-0006] Eisinger, W. , D. Ehrhardt , and W. Briggs . 2012. “Microtubules Are Essential for Guard‐Cell Function in Vicia and *Arabidopsis* .” Molecular Plant 5: 601–610. 22402260 10.1093/mp/sss002

[pld370063-bib-0007] Fukuda, M. , S. Hasezawa , N. Asai , N. Nakajima , and N. Kondo . 1998. “Dynamic Organization of Microtubules in Guard Cells of Vicia faba L. With Diurnal Cycle.” Plant & Cell Physiology 39: 80–86. 9517004 10.1093/oxfordjournals.pcp.a029293

[pld370063-bib-0008] Genova, M. , L. Grycova , V. Puttrich , et al. 2023. “Tubulin Polyglutamylation Differentially Regulates Microtubule‐Interacting Proteins.” EMBO Journal 42: e112101. 36636822 10.15252/embj.2022112101 PMC9975938

[pld370063-bib-0009] Hartweck, L. M. , C. L. Scott , and N. E. Olszewski . 2002. “Two *O*‐Linked *N*‐Acetylglucosamine Transferase Genes of *Arabidopsis Thaliana* l. Heynh. Have Overlapping Functions Necessary for Gamete and Seed Development.” Genetics 161: 1279–1291. 12136030 10.1093/genetics/161.3.1279 PMC1462182

[pld370063-bib-0010] Jacobsen, S. E. , K. A. Binkowski , and N. E. Olszewski . 1996. “SPINDLY, a Tetratricopeptide Repeat Protein Involved in Gibberellin Signal Transduction in *Arabidopsis* .” Proceedings of the National Academy of Sciences 93: 9292–9296. 10.1073/pnas.93.17.9292 PMC38635 8799194

[pld370063-bib-0011] Jacobsen, S. E. , and N. E. Olszewski . 1993. “Mutations at the SPINDLY Locus of *Arabidopsis* Alter Gibberellin Signal Transduction.” Plant Cell 5: 887–896. 8400871 10.1105/tpc.5.8.887 PMC160324

[pld370063-bib-0012] Ji, S. , J. G. Kang , S. Y. Park , J. Lee , Y. J. Oh , and J. W. Cho . 2011. “ *O*‐GlcNAcylation of Tubulin Inhibits Its Polymerization.” Amino Acids 40: 809–818. 20665223 10.1007/s00726-010-0698-9

[pld370063-bib-0013] Jiang, Y. , K. Wu , F. Lin , Y. Qu , X. Liu , and Q. Zhang . 2014. “Phosphatidic Acid Integrates Calcium Signaling and Microtubule Dynamics Into Regulating ABA‐Induced Stomatal Closure in *Arabidopsis* .” Planta 239: 565–575. 24271006 10.1007/s00425-013-1999-5

[pld370063-bib-0014] Kuromori, T. , M. Seo , and K. Shinozaki . 2018. “ABA Transport and Plant Water Stress Responses.” Trends in Plant Science 23: 513–522. 29731225 10.1016/j.tplants.2018.04.001

[pld370063-bib-0015] Li, X. , C. Lei , Q. Song , et al. 2023. “Chemoproteomic Profiling of *O*‐GlcNAcylated Proteins and Identification of *O*‐GlcNAc Transferases in Rice.” Plant Biotechnology Journal 21: 742–753. 36577688 10.1111/pbi.13991 PMC10037131

[pld370063-bib-0016] Lin, Z. , I. Gasic , V. Chandrasekaran , et al. 2020. “TTC5 Mediates Autoregulation of Tubulin via mRNA Degradation.” Science 367: 100–104. 31727855 10.1126/science.aaz4352 PMC6942541

[pld370063-bib-0017] Liu, W. , C. Wang , G. Wang , et al. 2019. “Towards a Better Recording of Microtubule Cytoskeletal Spatial Organization and Dynamics in Plant Cells.” Journal of Integrative Plant Biology 61: 388–393. 30226291 10.1111/jipb.12721

[pld370063-bib-0018] Mutanwad, K. V. , I. Zangl , and D. Lucyshyn . 2020. “The *Arabidopsis O*‐Fucosyltransferase SPINDLY Regulates Root Hair Patterning Independently of Gibberellin Signaling.” Development 147: 192039. 10.1242/dev.192039 PMC7567127 32928908

[pld370063-bib-0019] Qin, F. , K. S. Kodaira , K. Maruyama , et al. 2011. “SPINDLY, a Negative Regulator of Gibberellic Acid Signaling, Is Involved in the Plant Abiotic Stress Response.” Plant Physiology 157: 1900–1913. 22013217 10.1104/pp.111.187302 PMC3327212

[pld370063-bib-0020] Shrestha, R. , S. Karunadasa , T. S. Grismer , A. V. Reyes , and S. L. Xu . 2024. “SECRET AGENT *O*‐GlcNAcylates Hundreds of Proteins Involved in Diverse Cellular Processes in *Arabidopsis* .” Molecular & Cellular Proteomics 23: 100732. 38336175 10.1016/j.mcpro.2024.100732 PMC10979276

[pld370063-bib-0021] Sun, T. P. 2021. “Novel Nucleocytoplasmic Protein *O*‐Fucosylation by SPINDLY Regulates Diverse Developmental Processes in Plants.” Current Opinion in Structural Biology 68: 113–121. 33476897 10.1016/j.sbi.2020.12.013 PMC8222059

[pld370063-bib-0022] Walgren, J. L. E. , T. S. Vincent , K. L. Schey , and M. G. Buse . 2003. “High Glucose and Insulin Promote *O*‐GlcNAc Modificationof Proteins, Including α‐Tubulin.” American Journal of Physiology. Endocrinology and Metabolism 284: E424–E434. 12397027 10.1152/ajpendo.00382.2002

[pld370063-bib-0023] Wang, P. , S. Qi , X. Wang , et al. 2023. “The OPEN STOMATA1‐SPIRAL1 Module Regulates Microtubule Stability During Abscisic Acid‐Induced Stomatal Closure in *Arabidopsis* .” Plant Cell 35: 260–278. 36255272 10.1093/plcell/koac307 PMC9806620

[pld370063-bib-0024] Wang, Y. , Y. He , C. Su , R. Zentella , T. P. Sun , and L. Wang . 2020. “Nuclear Localized *O*‐Fucosyltransferase SPY Facilitates PRR5 Proteolysis to Fine‐Tune the Pace of *Arabidopsis* Circadian Clock.” Molecular Plant 13: 446–458. 31899321 10.1016/j.molp.2019.12.013 PMC7058189

[pld370063-bib-0025] Wu, J. , C. Lei , X. Li , et al. 2022. “Chemoproteomic Profiling of *O*‐GlcNAcylation in *Arabidopsis Thaliana* by Using Metabolic Glycan Labeling.” Israel Journal of Chemistry 63: e202200065.

[pld370063-bib-0026] Xu, S. L. , R. J. Chalkley , J. C. Maynard , et al. 2017. “Proteomic Analysis Reveals *O*‐GlcNAc Modification on Proteins With key Regulatory Functions in *Arabidopsis* .” Proceedings of the National Academy of Sciences 114: E1536–E1543. 10.1073/pnas.1610452114 PMC5338445 28154133

[pld370063-bib-0027] Yang, X. , and K. Qian . 2017. “Protein *O*‐GlcNAcylation: Emerging Mechanisms and Functions.” Nature Reviews. Molecular Cell Biology 18: 452–465. 28488703 10.1038/nrm.2017.22 PMC5667541

[pld370063-bib-0028] Yu, X. , X. Chen , M. Amrute‐Nayak , et al. 2021. “MARK4 Controls Ischaemic Heart Failure Through Microtubule Detyrosination.” Nature 594: 560–565. 34040253 10.1038/s41586-021-03573-5 PMC7612144

[pld370063-bib-0029] Zentella, R. , J. Hu , W.‐P. Hsieh , et al. 2016. “ *O*‐GlcNAcylation of Master Growth Repressor DELLA by SECRET AGENT Modulates Multiple Signaling Pathways in *Arabidopsis* .” Genes & Development 30: 164–176. 26773002 10.1101/gad.270587.115 PMC4719307

[pld370063-bib-0030] Zentella, R. , N. Sui , B. Barnhill , et al. 2017. “The *Arabidopsis O*‐Fucosyltransferase SPINDLY Activates Nuclear Growth Repressor DELLA.” Nature Chemical Biology 13: 479–485. 28244988 10.1038/nchembio.2320 PMC5391292

[pld370063-bib-0031] Zentella, R. , Y. Wang , E. Zahn , et al. 2023. “SPINDLY *O*‐Fucosylates Nuclear and Cytoplasmic Proteins Involved in Diverse Cellular Processes in Plants.” Plant Physiology 191: 1546–1560. 36740243 10.1093/plphys/kiad011 PMC10022643

