# Supplementary material for: SPY Interacts With Tubulin and Regulates Abscisic Acid‐Induced Stomatal Closure in Arabidopsis
Source: Plant Direct. 2025 Apr 1;9(4):e70063. doi: 10.1002/pld3.70063 (PMC11959150; doi:10.1002/pld3.70063)
Supplement: Supplementary file 1 — Figure S1 Identification of spy mutants. A Schematic structure of the SPY protein and the locations of the mutations in the mutants B. DNA electrophoretogram of the spy‐3 mutant (left) and sequencing result (right). Primers (F, R) were designed on both sides of the mutation site, and the target bands (240 bp) were amplified by PCR and then sequenced. C DNA electrophoretogram of the spy‐22 mutant and RT‐qPCR analysis (right). (M:marker molecular weight is 2000 bp, mutants 1 and 2 are homozygous lines). RT‐qPCR detection of SPY gene mRNA expression level was standardized with internal reference gene EF1‐α, and three independent biological replications were performed. Data are mean ± SEM. [file PLD3-9-e70063-s002.pdf]

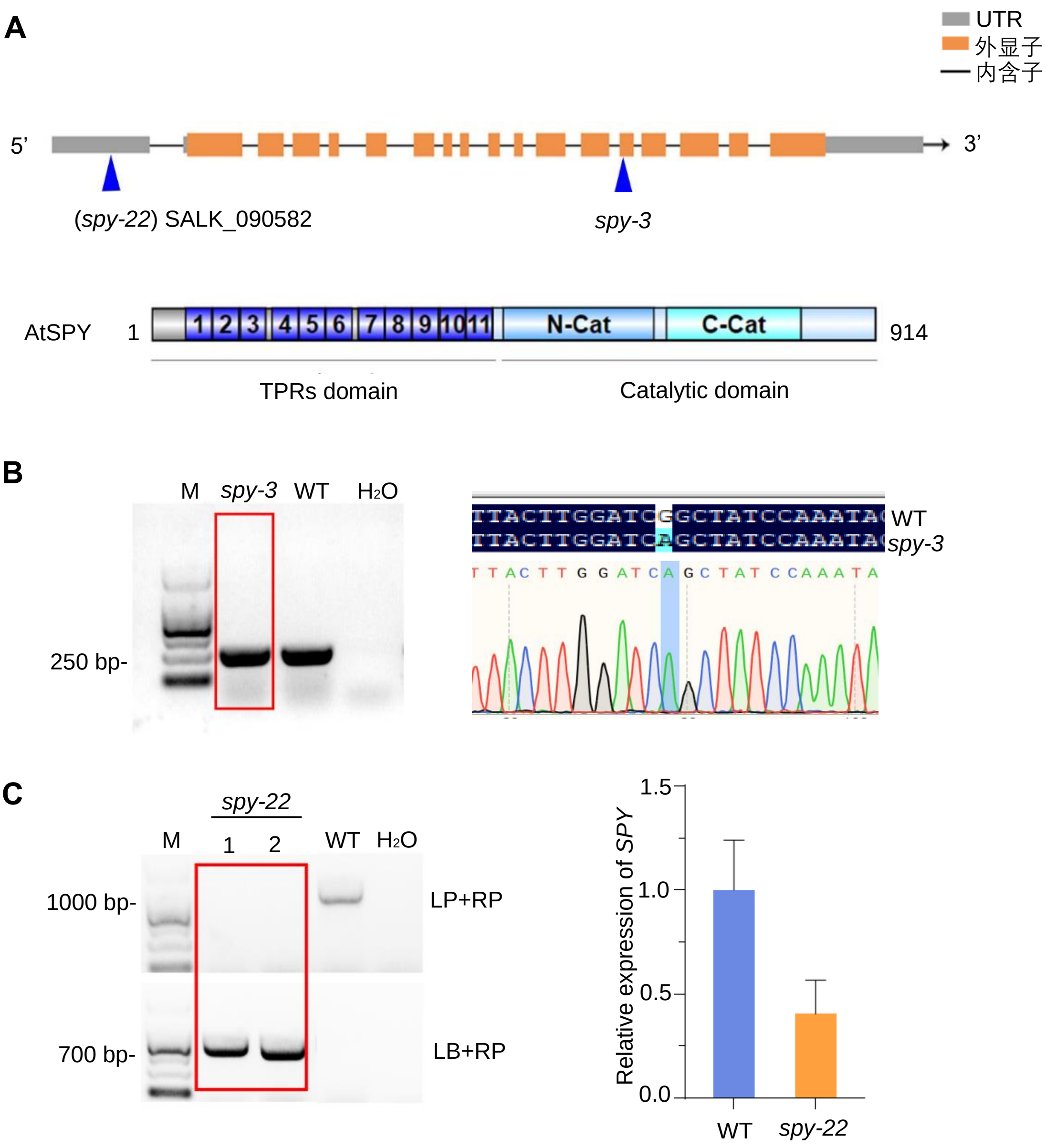

Fig S1 Identification of *spy* mutants. A Schematic structure of the SPY protein and the locations of the mutations in the mutants B. DNA electrophoretogram of the *spy-3* mutant (left) and sequencing result (right). Primers (F, R) were designed on both sides of the mutation site, and the target bands (240 bp) were amplified by PCR and then sequenced. C DNA electrophoretogram of the *spy-22* mutant and RT-qPCR analysis (right). (M: marker molecular weight is 2000 bp, mutants 1 and 2 are homozygous lines). RT-qPCR detection of *SPY* gene mRNA expression level was standardized with internal reference gene *EF1-α*, and three independent biological replications were performed. Data are mean ± SEM.
