# Supplementary material for: SPY Interacts With Tubulin and Regulates Abscisic Acid‐Induced Stomatal Closure in Arabidopsis
Source: Plant Direct. 2025 Apr 1;9(4):e70063. doi: 10.1002/pld3.70063 (PMC11959150; doi:10.1002/pld3.70063)
Supplement: Supplementary file 2 — Table S1 Primers for identification of spy‐22 (SALK_090582) mutants. Table S2 Primers for identification of spy‐3 mutants Table S3 Primers for construction of spy‐22 complemented lines Table S4 Primers for construction of TUA1 overexpressing transgenic plants Table S5 Primers for RT‐qPCR Table S6 Primers for yeast two‐hybrid assay Table S7 Primers for bimolecular fluorescence complementation (BiFC) assay Table S8 Primers for firefly luciferase complementation imaging (LCI) assay [file PLD3-9-e70063-s001.docx]

List of primers used in this study:

Supplemental Table S1 Primers for identification of *spy-22* (SALK_090582) mutants

| Primer name | Primer sequence (5’-3’) |
| --- | --- |
| LP | ATCTGAGTCGTTGCCTCTGTC |
| RP | TGTAATGTTGCACCTCAGCAG |
| LB | ATTTTGCCGATTTCGGAAC |

By using the three primers (LB+LP+RP) for SALK lines, WT (wild type, no insertion) should get a product of about 900–1100 bps (from LP to RP), HM (homozygous lines, insertions in both chromosomes) will get a band of 547–847 bps, and HZ (heterozygous lines, one of the pair chromosomes with insertion) will get both bands.

Supplemental Table S2 Primers for identification of *spy-3* mutants

| Primer name | Primer sequence (5’-3’) |
| --- | --- |
| *spy-3*-F | CCGGTTCAGGTGAGAGGATA |
| *spy-3*-R | CGAAACCCAAAATTGCATCT |

Supplemental Table S3 Primers for construction of *spy-22* complemented lines

| Primer name | Primer sequence (5’-3’) |
| --- | --- |
| SPY-CDS-F | TTTAGTTACAAAAAACTGCAGATGGTGGGACTGGAAGATGATACT |
| SPY-CDS-R | CATGGATCCTCTAGAGTCGACGCTAGTGGAGTCCATTCTCTTTGAG |
| SPY-Pro-F | ACGACGGCCAGTGCCAAGCTTTGATTGATTACCTTCGATGGTCAT |
| SPY-Pro-R | TCCTCTAGAGTCGACCTGCAGTTTTTTGTAACTAAAATCTTGTTCACCTT |

Supplemental Table S4 Primers for construction of TUA1 overexpressing transgenic plants

| Primer name | Primer sequence (5’-3’) |
| --- | --- |
| TUA1-F | TTTGGAGAGAACACGCTGCAGATGAGGGAGATCATTAGCAT |
| TUA1-R | CATGGATCCTCTAGAGTCGACATACTCATCGCCTTCTTCGT |

Supplemental Table S5 Primers for RT-qPCR

| Primer name | Primer sequence (5’-3’) |
| --- | --- |
| SPY-F | GAACTCACTGGTCATACGGC |
| SPY-R | ACAGTGGGCAAACCCGTAGT |
| EF-1α-F | TGAGCACGCTCTTCTTGCTTTCA |
| EF-1α-R | GGTGGTGGCATCCATCTTGTTACA |

Supplemental Table S6 Primers for yeast two-hybrid assay

| Primer name | Primer sequence (5’-3’) |
| --- | --- |
| AD-TUA1-F | GCCATGGAGGCCAGTGAATTCAGGGAGATCATTAGCATTCA |
| AD-TUA1-R | CAGCTCGAGCTCGATGGATCCATACTCATCGCCTTCTTCGT |
| AD-TUA4-F | GCCATGGAGGCCAGTGAATTCATGAGAGAGATCCTTCACATTC |
| AD-TUA4-R | CAGCTCGAGCTCGATGGATCCAGTCTCATAATCTCCCTCCTCT |
| AD-TUB5-F | GCCATGGAGGCCAGTGAATTCAGAGAGATCCTTCACATTC |
| AD-TUB5-R | CAGCTCGAGCTCGATGGATCCAGTCTCATAATCTCCCTCCT |
| BD-SPY-F | AGGCCGAATTCCCGGGGATCCTGGTGGGACTGGAAGATGAT |
| BD-SPY-R | CTAGTTATGCGGCCGCTGCAGTAGCTAGTGGAGTCCATTCT |

Supplemental Table S7 Primers for bimolecular fluorescence complementation (BiFC) assay

| Primer name | Primer sequence (5’-3’) |
| --- | --- |
| cYFP-TUA1-F | GAGAACACGGGGGACTCTAGAATGAGGGAGATCATTAGCAT |
| cYFP-TUA1-R | CTTCTGCTTGTCCATGGATCCATACTCATCGCCTTCTTCGT |
| nYFP-SPY-F | GAGAACACGGGGGACTCTAGAATGGTGGGACTGGAAGATGA |
| nYFP-SPY-R | GCCCTTGCTCACCATGGATCCGCTAGTGGAGTCCATTCTCT |

Supplemental Table S8 Primers for firefly luciferase complementation imaging (LCI) assay

| Primer name | Primer sequence (5’-3’) |
| --- | --- |
| cLUC-TUA1-F | TACGCGTCCCGGGGCGGTACCATGAGGGAGATCATTAGCAT |
| cLUC-TUA1-R | ACGAAAGCTCTGCAGGTCGACCTAATACTCATCGCCTTCTT |
| nLUC-SPY-F | ACGGGGGACGAGCTCGGTACCATGGTGGGACTGGAAGATGA |
| nLUC-SPY-R | ACGCGTACGAGATCTGTCGACGCTAGTGGAGTCCATTCTCT |
